# Occupational Exposure to Blood and Body Fluids among Medical Laboratory Science Students of the University of Health and Allied Sciences during Vocational Internship in the Volta Region of Ghana

**DOI:** 10.1155/2020/4878315

**Published:** 2020-06-01

**Authors:** Philip Apraku Tawiah, Kwabena Oppong, Emmanuel Sintim Effah, Albert Abaka-Yawson, Kingsley Arhin-Wiredu

**Affiliations:** ^1^Department of Pharmacognosy and Herbal Medicine, School of Pharmacy, University of Health and Allied Sciences, Ho, Ghana; ^2^Kumasi Centre for Collaborative Research in Tropical Medicine, Kumasi, Ghana; ^3^Mamprobi Polyclinic, Ghana Health Service, Mamprobi, Accra, Ghana; ^4^Department of Medical Laboratory Sciences, School of Allied Health Sciences, University of Health and Allied Sciences, Ho, Ghana; ^5^Municipal Health Directorate, Ghana Health Service, Sunyani, Ghana

## Abstract

Medical laboratory science students (MLSS), likewise health care workers (HCW), invariably get exposed to blood and body fluids (BBF) of patients. The degree of exposure of these students is even worsened due to their inexperience, which is usually revealed during their vocational training programme. This study therefore determined the prevalence of exposure to BBF and its risk factors among MLSS at the University of Health and Allied Sciences (UHAS). A cross-sectional survey was employed using simple random sampling to enrol 178 students into the study. The study was conducted from February 1 to March 31, 2018, after the annual vocational training programme completed in August 2017. Self-administered questionnaires based on the objectives of the study were given out to participants to complete after their consent was sought. Descriptive data were reported as absolute number with percentages, whereas bivariate and multiple logistic regressions were done to describe relationship between risk factors and exposure to BBF. The study findings revealed that, out of 178 MLSS that participated, 90 (50.6%) experienced at least one exposure to BBF. Also, work experience before university education increased the chances of exposure to BBF (AOR = 7.37, 95% CI = 1.22–44.43, *p*value = 0.029) compared with those with no experience. In contrast, adequate personal protective equipment (PPE) reduced the tendencies of exposure to BBF (AOR = 0.41, 95% CI = 0.20–0.88, *p* value = 0.023) compared with students who had insufficient PPE. The study showed high, 50.6% (95% CI: 43.0%–58.1%), exposure to BBF. Work history and sufficient PPE were the most significantly associated risk factors. In view of this, there is the need to promote training and education on exposure to BBF particularly among experienced students and also encourage health facilities to continue providing enough PPE for students during their annual obligatory vocational internship programmes.

## 1. Introduction

The exposure to BBF among HCW particularly MLSS is an inevitable public health burden that forms part of routine health care delivery and is significant in causing occasional infections and deaths [1]. These health care professionals are at risk of exposure to numerous virulent and deadly microorganisms comprising 26 viruses, 18 bacteria/Rickettsia, 13 parasites, and 3 yeasts that result mostly from exposure to BBF from patients [2, 3].

Additionally, the exposure to these pathogenic organisms usually results in three significant infections in the health care setting—human immunodeficiency virus (HIV), hepatitis B virus (HBV), and hepatitis C virus (HCV) [3]. Further, a study estimated that in the year 2000, 66,000 hepatitis B, 16,000 hepatitis C, and 200–500 HIV infections were acquired through exposure to BBF through splash of blood, needlestick, and cuts [4]. Unfortunately, among these three important blood-borne infections, only HBV has a vaccine [5].

According to the World Health Organization (WHO) in the year 2010, about 3 million people were at risk of exposure to blood-borne viruses and 90% of the exposure took place in low- or middle-income countries. Moreover, health care personnel including MLSS in developing countries are at serious risk of blood-transmitted diseases (HIV, HBV and HCV) because there is high prevalence in such areas, predominantly sub-Saharan Africa [6]. The Ghana Health Service (GHS) estimated that 30.8% of all laboratory-confirmed cases of HBV infection (blood-borne disease) were ascribed to the Volta region of Ghana [7], and this happens to be a part of the country where the MLSS of UHAS usually do their annual mandatory vocational internship programmes between the period of June and August.

As predicted in some studies, nurses, medical doctors, and laboratory technicians who are frequently exposed to BBF from patients are of HCW category [8, 9]. This makes MLSS prone to a higher risk of exposure because of their association with these groups of HCW, and inadequate experience in their line of work during their annual internship programmes also contributes to their risk. Furthermore, needlestick, splashes, cuts, and sharp injuries that expose HCW to blood and other body fluids have been associated with lack of experience in carrying out procedures, insufficient training, work overload, and fatigue [10, 11]. These experiences are characteristic among students embarking on vocational internship programmes.

Studies on prevalence of occupational exposure to BBF among health care workers and students in both developed [12, 13] and developing countries [14, 15] have been published. A study carried out among HCW in Serbia revealed that 29.6% of participants experienced at least one incidence that exposed them to BBF within the previous year [16]. A similar study conducted in Northwest Ethiopia also predicted a higher prevalence of 58.5% exposure to BBF in their lifetime [17]. A research work carried out among dental students during a period in their clinical course to determine the exposure of BBF found a high prevalence of 80%, whereas a comparable study carried out among nursing students after a clinical practicum that lasted for six months found 75.6% of them were exposed to BBF [18, 19]. A higher prevalence of 88.6% was found among nursing students during an internship year that lasted for three months in Assiut City, Egypt [20].

In Ghana, almost all studies published on exposure to BBF focused on HCW. A recent study among health personnel in a district hospital revealed that 67.5% of them were exposed to BBF in the last 12 months [21]. On the contrary, not many studies have been done among health care students, specifically none among MLSS. In view of this, the study determined the prevalence of exposure to BBF and its associated risk factors among MLSS embarking on vocational internship programmes in the Volta region of Ghana.

## 2. Materials and Methods

### 2.1. Study Design

A cross-sectional survey was employed using simple random sampling to recruit 178 MLSS in the UHAS into the study. The research work was conducted from February 1 to March 31, 2018. Self-administered questionnaires based on the objectives of the study were given out to participants to complete after their consent was sought.

### 2.2. Study Area

The research work was carried out in the UHAS. It is located at Ho in the Volta region of Ghana on 6.5739°N, 0.4410°E. The university happens to be one of the newest and youngest public universities in Ghana mandated to train health care personnel. Currently, it operates under six schools, namely School of Medicine, School of Nursing and Midwifery, School of Public Health, School of Basic and Biomedical Sciences, School of Pharmacy, and School of Allied Health Sciences. The School of Allied Health Sciences is situated on the premises of the Volta Regional Hospital, now Ho Teaching Hospital, a leading referral hospital in the region and a centre for the training of Allied Health Professionals. The School of Allied Health Sciences comprises of six departments including Department of Medical Laboratory Sciences (DMLS) where the study was carried out. The department offers both regular and sandwich/modular programmes for the award of a Bachelor's degree.

### 2.3. Sampling Procedure

Of 241 students, a total of 178 participants were enrolled for the study based on Cochran's formula, *Z*^2*∗*^*p* (1 − *p*)/*d*^2^, and prevalence of occupational exposure to BBF in a previous study among nursing students on internship [20]. A sample size of 178 was calculated using a prevalence (*p*) of 88.6%, 5% margin error (d), 95% confidence interval (Z) given as 1.96 and 15% attrition rate to cater for non-response. This total sample size was distributed depending on the class size of the year groups, and the simple random sampling method was then used to recruit participants from each year into the study. A 100% response rate was achieved since all the students who were recruited consented and partook in the study.

#### 2.3.1. Inclusion Criteria

Continuing students who were in their 2^nd^, 3^rd^, or 4^th^ year and had completed the annual compulsory vocational training programme held within the period of June to August 2017 were eligible for the study.

#### 2.3.2. Exclusion Criteria

Fresh students who were in their 1^st^ year and had not completed the annual compulsory vocational training programme held within the interval of June–August 2017 were ineligible and were not recruited for the study.

### 2.4. Data Collection Technique

Self-administered closed-ended structured questionnaires were used to gather relevant data on the study objectives and research questions. This information included sociodemographic characteristics, standard precautionary measures, HBV vaccination status, as well as routes of exposure to BBF based on earlier investigations [18, 20, 21]. The questionnaires were completed by eligible participants and collected before and after lecture periods within the two-month duration of data collection.

### 2.5. Data Processing and Analysis

Data from the completed questionnaires were then entered into Microsoft Excel and imported into Stata, version 15 (64 bit), for editing and analysis. Descriptive statistics such as frequency and proportion were used to analyse the demographic factors, risk factors, and exposure to BBF. Chi-square test and Fisher's exact test were used to test for the association between risk factors and exposure to BBF, based on a statistical significance at 95% confidence interval. Crude odds ratios, adjusted odds ratios, 95% confidence intervals, and *p*values were calculated using bivariate and multiple logistic regression to describe the relationship between exposure to BBF and their associated risk factors. The variables with an observed association of *p* < 0.05 were noted and considered significant.

#### 2.5.1. Categorization of Exposure Status

The categorization of participants' exposure status was based on incidence of four routes of exposure to BBF during the vocational internship period held between June and August 2017. These were needlestick, sharp injuries, splash, and torn gloves. Participants who were not exposed to blood in any of the four routes of exposure during the last vocational training programmes were classified as nonexposed, whereas others who experienced at least one of the ways of exposure were grouped as exposed.

### 2.6. Ethical Consideration

Approval of the study protocol was obtained from the Ethical Review Committee of the Ghana Health Service (GHS-ERC) before commencement of the study. Permission was sought from the Department of Medical Laboratory Sciences (DMLS) of the UHAS before the data collection. Study participants were briefed about the purpose, risk, and benefits of the study before appending their signature on the consent form to take part in the survey.

## 3. Results

### 3.1. Sociodemographic Characteristics of Study Participants

A total of 178 MLSS were enrolled into the study. As depicted, 139 (78.1%) of these participants were males, and the majority 118 (66.3%) were 20–24 years of age. Most of the students, 166 (93.3%), were Christians. Most of the participants, 165 (92.7%), were not married. With respect to residence, 143 (80.3%) lived outside campus. In addition, more than half, 91 (51.1%), of students were in their 2^nd^ year, whereas 64 (36.0%) and 23 (12.9%) were in their 3^rd^ and 4^th^ year, respectively. A considerable number of students, 155 (87.1%), had no working experience before university education. During the time of the study, a majority of students, 105 (59.0%), had embarked on vocational training for less than two months (Table 1).

### 3.2. Prevalence of Exposure to BBF according to Sociodemographic Characteristics

A higher prevalence (51.3%) of exposure to BBF was found among students who were females. Also, a greater prevalence (53.9%) was reported among students who were married, whereas 51.1% was reported among students who did not stay on campus. In addition, the age range “20–24,” 4^th^ year group, and students who did 2–4 months' vocational training recorded the highest exposure to BBF as 54.2%, 56.5%, and 61.5%, respectively. Also, students who had worked before university education had a higher exposure prevalence of 60.9% compared with those who never worked (Table 1).

### 3.3. Exposure Status of Study Participants

**Figure 1:**
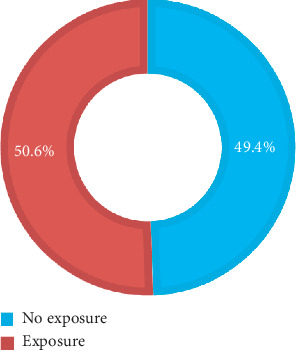
Exposure status of study participants.

A little more than half, 50.6%, of participants were exposed to BBF while undergoing the 2017 vocational training programme (Figure 1).

### 3.4. Occupational Exposure to BBF among Study Participants

**Figure 2:**
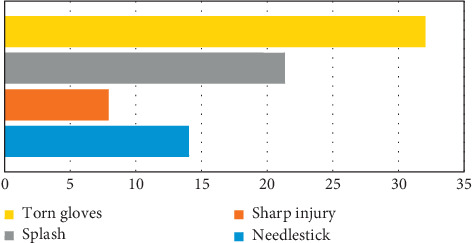
Routes of occupational exposure to BBF among participants.

Of the participants surveyed, 57 (32.0%) experienced torn gloves, 38 (21.3%) experienced splash of BBF, 25 (14.0%) experienced needlestick, and 14 (7.9%) had some form of sharp-related injuries (Figure 2).

### 3.5. Association between Risk Factors and Exposure to BBF Status

The only risk factor that showed a significant association with exposure to BBF status was the sufficient PPE to students on vocational internship programmes (Pearson chi^2^ = 5.3, *p*value = 0.021) (Table 1).

### 3.6. Bivariate and Multiple Logistic Regression Analysis on Exposure to BBF

Students who had work experience in a medical laboratory before pursuing university education had increased the odds of exposure to BBF by more than 7 times (AOR = 7.37, 95% CI = 1.22–44.43, *p*value = 0.029) compared with those who had no work experience. On the contrary, sufficient PPE to students decreased the odds of exposure to BBF by almost 59% (AOR = 0.41, 95% CI = 0.20–0.88, *p*value = 0.023) compared with students who did not have access to sufficient PPE (Table 2).

## 4. Discussion

MLSS and all other HCW inevitably get exposed to BBF of patients through needlestick, sharp injuries, and mucocutaneous contamination [22]. The extent of exposure of these students is even heightened due to their inexperience during their annual compulsory vocational training programmes [10]. As a result, it was imperative to study and discuss the possible risk factors of exposure to BBF to avert impending exposure in future vocational internships.

The present study depicted that the majority (87.1%) of participants had no work experience prior to the annual vocational internship programme held in 2017. This finding was in line with a study among dental and nursing students where the majority (83.6%) had no or less than 6 months experience [20]. Although the study revealed that work history was not significantly associated with exposure status, inadequate experience has been suggested as an increased risk of occupational injury among students carrying out invasive medical procedures [20].

Our study found that only 74.2% of participants had access to sufficient PPE. This result was in contrast to the study by Yasin et al. [17] in Ethiopia where 77.0% of HCW complained of not having adequate PPE over the past one year. The difference in results may be due to variations in the strength of health care system, supply chain, and finances of health care facilities. The significant association observed between sufficient PPE and exposure status solidifies the statement made by the WHO amidst the coronavirus disease 19 (COVID-19) pandemic that HCW rely solely on PPE for protection against infection from patients [23].

Again, it is evident in our study that 56.4% of MLSS had received vaccination against hepatitis B virus infection. This result was not in coherent with a study in Kenya among HCW that revealed 40.0% hepatitis B vaccination coverage [24]. However, our finding was comparable with a study that predicted 55.3% hepatitis B vaccination status among HCW [17]. Although our study showed a little over 50% hepatitis B vaccination coverage, it does not meet the WHO-recommended coverage of 100%. In the context of this study, the possible causes of low coverage of hepatitis B vaccination may comprise the two major factors—high cost of vaccine and lack of policy to make hepatitis B vaccination compulsory for health care students.

The current study showed that 52.3% of study participants had received training on infectious diseases and prevention that was organized by their host health care facilities prior to the start of their vocational training programme. This result was not consistent with a study conducted by [25] that found 48.8% of participants had received training on infectious diseases and prevention. The dissimilarity in results may be due to the priority management of health care facilities places on training focused on infectious diseases and prevention and in some cases the lack of funds to organize continuous training programmes for HCW.

Further, our results showed that almost all (99.4%) of participants used gloves one way or the other in their line of work, yet, of all the routes of exposure, torn gloves was experienced by most students with a prevalence of 32.0%. This result was similar to a study where participants experienced 28.9% of glove breakage [26]. Glove breakage can be associated with sudden movement of patients during sample collection and waste disposal [27]. Moreover, the low quality of gloves is also known to contribute to this situation. This even confirms the reason why most HCW practice double gloving when performing a high risk procedure. Additionally, torn gloves is mostly experienced in the process of wearing and removing, and the degree of exposure is worse when removing it after a procedure has been done. This certainly calls for education on wearing and removal of gloves among HCW especially health care students.

Our current study predicted that 50.6% of participants were exposed to BBF; this result is similar to studies conducted in Durban, Iran, and Egypt where 55.0%, 53.4%, and 51.3% of medical interns, health care professionals, and dental students, respectively, were exposed to BBF in the past one year [20, 28, 29]. Nevertheless, the finding of this study was lower than BBF exposure levels, 88.6% and 75.6% predicted among nursing students in some studies [18, 20]. The differences in findings may be due to lack of training on infectious diseases, its control and exposure, duration of internship/vocational training programmes, nonadherence to safety precautionary measures, insufficient PPE and supporting facilities, and duration of exposure to BBF [17, 20].

With reference to our findings, work experience increased the exposure to BBF among students who worked before pursing university education more than 7 times (AOR = 7.37, 95% CI = 1.22–44.43, *p*value = 0.029) compared with those who had no work experience. The evidence from our study was similar to a study among HCW where participants who had more than 10 years' experience had over 4 times (AOR = 4.13, 95% CI = 1.56–10.91) risk of exposure compared with those who had less than two years' experience [30]. In our Ghanaian health settings, it is not common to find experienced HCW who adhere to all precautionary measures. However, this attitude of experienced HCW may be influenced by the lack of PPE in our health facilities and forgetfulness that usually comes into play when handling medical emergencies.

Finally, our study revealed that for participants who had sufficient PPE, the odds of exposure to BBF reduced by almost 59% (AOR = 0.41, 95% CI = 0.20–0.88, *p*value = 0.023) compared with students who did not had inadequate PPE at their disposal. This discovery is coherent to the study by Yenesew and Fekadu in which shortage of PPE increased the odds of exposure to BBF by approximately 2 times (COR = 1.75, 95% CI = 1.09–2.79) compared with those who did not experience shortage of PPE in their health facility [27]. The use of PPE from this study and other studies have proven to be the surest way of preventing exposure to BBF and its availability counts in the prevention of these exposures.

## 5. Limitations of the Study

Although students were asked to remember the exposure they encountered during their last vocational internship programme that was held between June and August 2017, approximately eight (8) months ago, there is still possibility of recall bias among these students and this might have contributed to distortion in the prevalence of exposure to BBF. As well, generalization of the results of this study is limited since participants were drawn from only one institution. It is probable that a large sample size of participants from different institutions may have presented a similar but different picture.

## 6. Conclusion

The study revealed that 50.6% (95% CI: 43.0%–58.1%) of MLSS experienced at least one exposure to BBF during the vocational internship. Work history before pursuing university education and sufficient PPE were the most significantly associated risk factors of exposure to BBF. Torn gloves was the most prevalent route of exposure to BBF among needlestick, splashes of BBF, and sharp-related injuries.

## Figures and Tables

**Table 1:** Chi-square analysis of factors influencing exposure to BBF.

Variable	Frequency, *n* = 178 (100.0%)	No exposure, *n* = 88 (49.4%)	Exposure, *n* = 90 (50.6%)	Chi-square	*p* value
*Gender*				0.0	0.919
Female	39 (21.9)	19 (48.7)	20 (51.3)		
Male	139 (78.1)	69 (49.6)	70 (50.4)		

*Age in years*				2.8	0.417
15–19	14 (7.9)	9 (64.3)	5 (35.7)		
20–24	118 (66.3)	54 (45.8)	64 (54.2)		
25–29	31 (17.4)	18 (58.1)	13 (41.9)		
>29	15 (8.4)	7 (46.7)	8 (53.3)		

*Work history*				1.2	0.289
Never worked	155 (87.1)	79 (51.0)	76 (49.0)		
Ever worked	23 (12.9)	9 (39.1)	14 (60.9)		

*Year group*				0.5	0.767
2^nd^ year	91 (51.1)	47 (51.7)	44 (48.3)		
3^rd^ year	64 (36.0)	31 (48.4)	33 (51.6)		
4^th^ year	23 (12.9)	10 (43.5)	13 (56.5)		

*Vocational*				3.6	0.293^*a*^
<2 months	105 (59.0)	57 (54.3)	48 (45.7)		
3–4 months	52 (29.2)	20 (38.5)	32 (61.5)		
5–6 months	15 (8.4)	8 (53.3)	7 (46.7)		
>6 months	6 (3.4)	3 (50.0)	3 (50.0)		

*Use of gloves*				1.0	0.506^*a*^
No	1 (0.6)	0 (0.0)	1 (100.0)		
Yes	177 (99.4)	88 (49.7)	89 (50.3)		

*Disinfection*				0.0	1.000^*a*^
No	2 (1.1)	1 (50.0)	1 (50.0)		
Yes	176 (98.9)	87 (49.4)	89 (50.6)		

*Training on ID*				0.8	0.364
No	85 (51.98)	39 (45.9)	46 (54.1)		
Yes	93 (52.25)	49 (52.7)	44 (47.3)		

*Vaccinated*				0.1	0.764
No	101 (56.7)	51 (50.5)	50 (49.5)		
Yes	77 (43.3)	37 (48.1)	40 (51.9)		

*Sufficient PPE*				5.3	0.021^*∗*^
No	46 (25.8)	16 (34.8)	30 (65.2)		
Yes	132 (74.2)	72 (54.5)	60 (45.5)		

All *p*values were calculated using the chi-square test except the ones denoted by ^*a*^ which were using Fisher's exact test. *p*values denoted by ^*∗*^ are significant at *p* < 0.05.

**Table 2:** Bivariate and multivariate logistic regression analysis of factors influencing exposure to BBF.

Variable	Frequency	No exposure	Exposure	*p*value	COR	*p* value	AOR
	*n* = 178 (100.0%)	*n* = 88 (49.4%)	*n* = 90 (50.6%)		OR (95% CI)		OR (95% CI)
*Gender*							
Female	39 (21.9)	19 (48.7)	20 (51.3)	0.919	1.00		1.00
Male	139 (78.1)	69 (49.6)	70 (50.4)		0.96 (0.47–1.96)	0.779	1.12 (0.49–2.56)

*Age in years*							
15–19	14 (7.9)	9 (64.3)	5 (35.7)		1.00		1.00
20–24	118 (66.3)	54 (45.8)	64 (54.2)	0.197	2.13 (0.67–6.75)	0.528	1.52 (0.41–5.58)
25–29	31 (17.4)	18 (58.1)	13 (41.9)	0.694	1.30 (0.35–4.80)	0.479	0.55 (0.11–2.83)
>29	15 (8.4)	7 (46.7)	8 (53.3)	0.343	2.06 (0.46–9.14)	0.294	0.27 (0.02–3.12)

*Year group*							
2^nd^ year	91 (51.1)	47 (51.7)	44 (48.3)		1.00		1.00
3^rd^ year	64 (36.0)	31 (48.4)	33 (51.6)	0.694	1.14 (0.60–2.16)	0.392	0.65 (0.24–1.74)
4^th^ year	23 (12.9)	10 (43.5)	13 (56.5)	0.485	1.39 (0.55–3.49)	0.292	2.10 (0.53–8.40)

*Work history*							
Never worked	155 (87.1)	79 (51.0)	76 (49.0)		1.00		1.00
Ever worked	23 (12.9)	9 (39.1)	14 (60.9)	0.292	1.62 (0.66–3.96)	0.029^*∗*^	7.37 (1.22–44.43)

*Vocational*							
<2 months	105 (59.0)	57 (54.3)	48 (45.7)		1.00		1.00
3–4 months	52 (29.2)	20 (38.5)	32 (61.5)	0.063	1.90 (0.96–3.74)	0.073	2.45 (0.92–6.58)
5–6 months	15 (8.4)	8 (53.3)	7 (46.7)	0.945	1.03 (0.35–3.07)	0.303	0.44 (0.10–2.06)
>6 months	6 (3.4)	3 (50.0)	3 (50.0)	0.838	1.18 (0.23–6.16)	0.945	1.08 (0.14–8.45)

*Vaccinated*							
No	101 (56.74)	51 (50.5)	50 (49.5)		1.00		1.00
Yes	77 (43.26)	37 (48.1)	40 (51.9)	0.747	1.10 (0.60–2.00)	0.932	1.02 (0.53–2.00)

*Training on ID*							
No	85 (51.98)	39 (45.9)	46 (54.1)		1.00		1.00
Yes	93 (52.25)	49 (52.7)	44 (47.3)	0.365	0.76 (0.42–1.37)	0.689	0.88 (0.46–1.68)

*Sufficient PPE*							
No	46 (25.8)	16 (34.8)	30 (65.2)		1.00		1.00
Yes	132 (74.2)	72 (54.5)	60 (45.5)	0.02^*∗*^	0.44 (0.22–0.89)	0.023^*∗*^	0.41 (0.20–0.88)

*p* values denoted by ^*∗*^ are significant at<0.05.

## Data Availability

Research data will be made available upon request.

## Abbreviations

AOR: Adjusted odds ratio
BBF: Blood and body fluids
CI: Confidence interval
COR: Crude odds ratio
HCW: Heath care workers
MLSS: Medical laboratory science students
PPE: Personal protective equipment
UHAS: University of Health and Allied Sciences
WHO: World Health Organization

## Additional Points

*Definition of Operational Terms.* Needlestick: wounds or injuries caused by needles that accidentally puncture the skin during health care medical procedures. Sharp injury: exposure events that result when medical sharps such as scalpels, broken glass, and broken capillary tube penetrate the skin.
